# Detecting the extent of control over selection bias relating to oral health and otorhinolaryngology: cross-sectional study

**DOI:** 10.1590/1516-3180.2019.0458.R1.04022020

**Published:** 2020-06-22

**Authors:** Christiane Alves Ferreira, Álvaro Nagib Atallah, Carlos Alfredo de Salles Loureiro

**Affiliations:** I MSc. Doctoral Researcher within Health Sciences, Department of Internal Medicine and Therapeutics and Evidence-Based Healthcare, Universidade Federal de São Paulo - Escola Paulista de Medicina (UNIFESP-EPM), São Paulo (SP), Brazil.; II MD, PhD. Titular Professor, Department of Internal Medicine and Therapeutics and Evidence-Based Healthcare, and Director, Brazilian Cochrane Center, Universidade Federal de São Paulo - Escola Paulista de Medicina (UNIFESP-EPM), São Paulo (SP), Brazil.; III MD. Doctoral Student, Department of Internal Medicine and Therapeutics and Evidence-Based Healthcare, Universidade Federal de São Paulo - Escola Paulista de Medicina (UNIFESP-EPM), São Paulo (SP), Brazil.

**Keywords:** Bias, Randomized controlled trial [publication type], Random allocation, Oral health, Selection bias, Cross-sectional studies, Dentistry, ENT diseases, Low risk of bias, Quality of randomized controlled trial

## Abstract

**BACKGROUND:**

The authors of randomized controlled trials will usually claim that they have met the randomization process criterion. However, sequence generation schemes differ and some schemes that are claimed to be randomized are not genuinely randomized. Even less well understood, and often more difficult to ascertain, is whether the allocation was really concealed.

**OBJECTIVE:**

To detect the extent of control over selection bias, in a comparison between two Cochrane groups: oral health and otorhinolaryngology; and to describe the methods used to control for this bias.

**DESIGN AND SETTING:**

Cross-sectional study conducted in a public university in São Paulo, Brazil.

**METHODS:**

The risk of selection bias in 1,714 records indexed in Medline database up to 2018 was assessed, independent of language and access. Two dimensions implicated in the allocation were considered: generation of the allocation sequence; and allocation concealment.

**RESULTS:**

We included 420 randomized controlled trials and all of them were evaluated to detect selection bias. In the sample studied, only 28 properly controlled the selection bias. Lack of control over selection bias was present in 80% of the studies evaluated in both groups.

**CONCLUSION:**

The two groups were similar regarding control over selection bias. They are also similar to the methods used. The dimension of allocation concealment appears to be a limiting factor with regard to production of randomized controlled trials with low risk of selection bias. The quality of reporting in studies on oral health and otorhinolaryngology is suboptimal and needs to be improved, in line with other fields of healthcare.

## INTRODUCTION

Randomized controlled trials (RCTs) are considered to be a powerful research design for evaluating the effects of healthcare interventions. They constituted one of the most important scientific advances during the 20^th^ century. Through using such trials, researchers have the assurance that the differences found between the groups evaluated truly result from the effectiveness of the intervention, given that the allocation is random, i.e. there is an equal distribution of prognoses between the groups.^1^^,^^2^^,^^3^

The controls over the allocation implementation process include generation of a random allocation sequence and simultaneous allocation concealment.^4^^,^^5^ It is fundamentally important that the investigators should not be capable of anticipating the allocation of the next participants. Absence of controls over the allocation process is a major barrier to internal validity, because this allows the researcher to predict the participants’ allocation at the recruitment stage. Even when bias signals are minimal, systematic differences in prognosis can be expected between the groups that will be compared. In particular, selection bias may compromise any randomized experiment in which the enrollment of subjects is sequential and the administration of treatments is unmasked.^6^^,^^7^^,^^8^^,^^9^

The authors of RCTs will usually claim that they have met the randomization process criterion, in the title of the article or in the abstract. However, sequence generation schemes differ, and some schemes that are claimed to be randomized are not genuinely randomized.^10^^,^^11^^,^^12^^,^^13^

Even less well understood, and often more difficult to ascertain, is whether the allocation was really concealed. Allocation concealment is actually part of the randomization process and, while distinct from the method used to generate the randomized sequence, is essential to the success of randomization.^13^^,^^14^^,^^15^

The idea of comparing data from different fields within healthcare is not new.^16^^,^^17^^,^^18^ However, today, there is a tendency for each research group only to evaluate data from their own field.^19^^,^^20^^,^^21^^,^^22^^,^^23^^,^^24^^,^^25^^,^^26^^,^^27^^,^^28^^,^^29^^,^^30^^,^^31^^,^^32^^,^^33^^,^^34^^,^^35^^,^^36^^,^^37^^,^^38^ True randomized controlled trials allow healthcare providers to make informed inferences about the validity of these trials.

## OBJECTIVE

The aims of this study were to detect the extent of control over selection bias through comparing different fields within healthcare: otorhinolaryngology and oral health; and to describe the methods used to control for this bias.

## METHODS

This was a cross-sectional study in which the risk of selection bias of randomized controlled trials indexed in the Medline database was assessed.

Primary studies with an RCT design in the fields of otorhinolaryngology (i.e. ear, nose and throat (ENT) diseases) and oral healthcare that were published in journals and indexed in the Medline database were eligible, independent of language and access. A random sample was taken from these studies. These two fields of healthcare were chosen because of the similar numbers of Cochrane systematic reviews that have been published, for reasons of their anatomical and functional proximity and because they share healthcare problems.

Since it was not possible to conduct a cohort study to evaluate the methods of randomized controlled trials, we chose to conduct two cross-over studies at two different time points: 2011-2013 and 2018-2020. Data from this second period have already begun to be collected and as soon as finalized will be compared and published. Thus, initially, approximately 755 potential RCTs in the oral health group and 959 potential RCTs in the ENT group were retrieved.

### Sample size

The sample calculation was performed taking a margin of error of 5%, a difference between groups of 30% and a confidence interval of 95%, in the light of our working hypothesis. It was found that analysis on approximately 200 articles in each field would be necessary. A total of 420 RCTs were included, comprising 214 on ENT and 206 on oral health.

### Types of data and methods (variables and bias)

To evaluate the allocation process, the Cochrane Collaboration risk-of-bias tool was used.^9^ Two dimensions implicated in the allocation were considered: generation of the allocation sequence; and allocation concealment.

The variable “risk of bias” was segmented into three possible responses: a) low risk of bias, meaning that the author controlled the entire allocation process, comprising random generation and allocation concealment, and therefore that this was an RCT with low risk of selection bias; b) uncertain risk of bias, meaning that the author did not report the allocation process comprising random generation and concealment of the allocation, or reported it in such a way that it was impossible to know for sure what the real situation of the allocation and concealment was; and c) high risk of bias, meaning that the author reported using an incorrect method for the allocation process.

### Search methods for identifying studies (data sources)

**Figure 1.**
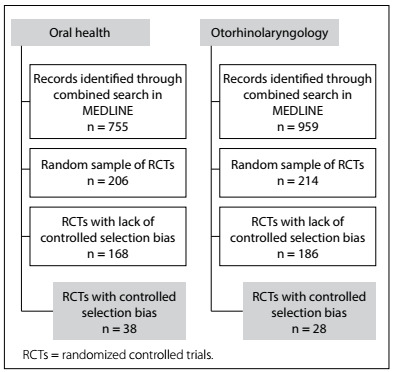
Flowchart of search.

To identify RCTs in the ENT group, an electronic search strategy recommended by the Cochrane Ear, Nose and Throat Disorders Group was used, with the following search terms: ear nose throat; general ENT and head-and-neck cancer; nose and adenoids, pharynx, larynx and upper respiratory tract infection (URTI); salivary glands; and skull base and neck. This was combined with the Cochrane highly sensitive search strategies for identifying randomized trials in Medline (Figure 1). The same approach was used for identifying RCTs in the oral health group (Figure 1).

### Data extraction and management

To assess the risk of the RCTs, two independent reviewers classified the studies, to ensure that these trials could be replicated. The first was a medical researcher (CAF) who was an expert on methodology; and the second was a pharmacist who was an expert on epidemiology (VA). Disagreements were discussed with a third researcher, who was an expert on methodology, to establish a consensus (CASL).

### Statistical processing of data

The statistical analyses were performed using the SPSS 19.0 software for Windows. The variable “risk of bias” was taken to be the ordinal and each group was taken to be the categorical variable. To ascertain differences between the groups within each dimension of the risk of bias, nonparametric test were used (Pearson chi-square and Mantel-Haenszel). The odds ratio (OR) was used to represent the chances between the groups evaluated. The significance level used in the tests was 5% (alpha = 0.05), and tests with P-values less than 5% (P < 0.05) were taken to be statistically significant.

## RESULTS

The Cochrane strategy combined with the key words in the field of oral health recovered 755 records. From this universe, a sample of 206 RCTs was taken. All of these RCTs were evaluated to detect selection bias. In the sample studied, only 38 RCTs properly controlled selection bias (Figure 1).

The Cochrane strategy combined with the key words in the field of ENT recovered 959 records. From this universe, a sample of 214 RCTs was taken. All of these RCTs were evaluated to detect selection bias. In the sample studied, only 28 RCTs properly controlled selection bias (Figure 1).

The level of agreement between the reviewers was measured using the kappa statistical test. The result was satisfactory (kappa = 0.71).

Table 1 presents the classification of the studies regarding the dimension of generation of the allocation sequence in the fields. In both groups, 43% of the studies presented low risk of bias (89 on oral health and 92 on ENT). No significant differences in the distribution of the studies were observed in comparing between the fields (P > 0.943). More than half of the RCTs in the two groups were classified as presenting uncertain risk of bias (108 on oral health and 114 on ENT) and approximately 4% in the two groups presented high risk of bias (9 on oral health and 8 on ENT).

**Table 1.** Classification of risk of bias in 420 studies regarding the dimension of random sequence generation in the Cochrane oral health and otorhinolaryngology (ear, nose and throat, ENT) groups

		Groups		Total	Pearson chi-square test
		Oral health	ENT		
Random sequence generation	Low risk of bias				0.943
	Frequency	89	92	181	
	% within allocation Sequence generation	49.20%	50.80%	100.00%	
	% within subject	43.20%	43.00%	43.10%	
	% of total	21.20%	21.90%	43.10%	
	Uncertain risk of bias				
	Frequency	108	114	222	
	% within allocation Sequence generation	48.60%	51.40%	100.00%	
	% within subject	52.40%	53.30%	52.90%	
	% of total	25.70%	27.10%	52.90%	
	High risk of bias				
	Frequency	9	8	17	
	% within allocation Sequence generation	52.90%	47.10%	100.00%	
	% within subject	4.40%	3.70%	4.00%	
	% of total	2.10%	1.90%	4.00%	
	Total				
	Frequency	206	214	420	
	% within allocation Sequence generation	49.00%	51.00%	100.00%	
	% within subject	100.00%	100.00%	100.00%	
	% of total	49.00%	51.00%	100.00%	

Table 2 shows the classification of the studies with regard to allocation concealment in the fields of ENT and oral health. For this dimension, 26% of the RCTs on oral health were considered to present low risk of bias and 19% on ENT (54 on oral health and 40 on ENT). No significant differences in the distribution of the studies were observed in comparing between the two groups (P > 0.124). In addition, 73% of the oral health RCTs and 80% of those within ENT were classified presenting uncertain risk of bias (151 on oral health and 171 on ENT). Less than 2% in both groups presented high risk of bias (one on oral health and three on ENT).

**Table 2.** Classification of risk of bias in 420 studies regarding the dimension of allocation concealment in the Cochrane oral health and otorhinolaryngology (ear, nose and throat, ENT) groups

		Groups		Total	Pearson chi-square test
		Oral health	ENT		
Allocation conceal-ment	Low risk of bias				0.124
	Frequency	54	40	94	
	% within concealment allocation	57.40%	42.60%	100.00%	
	% within subject	26.20%	18.70%	22.40%	
	% of total	12.90%	9.50%	22.40%	
	Uncertain risk of bias				
	Frequency	151	171	322	
	% within concealment allocation	46.90%	53.10%	100.00%	
	% within subject	73.30%	79.90%	76.70%	
	% of total	36.00%	40.70%	76.70%	
	High risk of bias				
	Frequency	1	3	4	
	% within concealment allocation	25.00%	75.00%	100.00%	
	% within subject	0.49%	1.40%	1.00%	
	% of total	0.24%	0.70%	1.00%	
	Total				
	Frequency	206	214	420	
	% within concealment allocation	49.00%	51.00%	100.00%	
	% within subject	100.00%	100.00%	100.00%	
	% of total	49.00%	51.00%	100.00%	

Table 3 presents the percentages of the RCTs that were controlled for selection bias in the two groups. Lack of control over selection bias was present in 80% of the studies evaluated in the two groups. Only 38 (18%) of the studies evaluated within oral health and 28 (13%) within ENT were RCTs with control over selection bias or with low risk of selection bias. The odds ratio (OR) was 1.45, with a range from 0.85 to 2.47, which was therefore not significant. The chance that a study would be an RCT with low risk of selection bias was almost 1.5 times greater in the oral healthcare group than in the ENT group.

**Table 3.** Frequency of randomized controlled trials with control over selection bias within the fields of ear, nose and throat (ENT) diseases and oral health

Risk of selection bias	Groups			Value			Mantel-Haenszel test
	Oral health	ENT	Total	OR	95% CI		
Low risk of bias							
Count	37	28	65				
% within subject	18%	13%	16%				
% of total	9%	7%	16%				
Uncertain or high risk of bias							
Count	169	186	355	1.45	0.85	2.47	0.169
% within subject	82%	87%	85%				
% of total	40%	44%	85%				
Total							
Count	206	214	420				
% within subject	100%	100%	100%				
% of total	49%	51%	100%				

CI = confidence interval; OR = odds ratio.

Different methods were used for generating the allocation process. Most of the authors used unrestricted randomization to generate the allocation sequence, like computer programs or tables or lists of random numbers. Flipping a coin was used only in the oral health group. Central randomization was used only in the ENT group. Some authors used restricted randomization, like randomization in blocks: this was seen both in the ENT group and in the oral health group. Only three authors used minimization in the ENT group and only two used minimization in the oral health group. Only one author used random allocation by drawing lots, and this was within oral health (Figure 1).

Most of the authors used sealed numbered envelopes to accomplish allocation concealment. Only two studies used central allocation to achieve concealment in the ENT group and one study in the oral health group (Figure 1).

## DISCUSSION

Many study projects may control for large numbers of types of bias, but the means used for adequately applying the allocation process in order to control selection bias is precisely the feature that distinguishes RCTs from other types of study project.^9^

In the present study, it was found that selection bias was possible in most of the studies evaluated in both fields. The dimension of allocation concealment appears to be a limiting factor with regard to production of RCTs with low risk of bias, thus representing the main barrier against production of RCTs with control over selection bias. Only 16% of the studies were truly RCTs or enabled control over selection bias.

The two groups were identical regarding control over selection bias and, thus, this appears not to be a condition relating only to the field of oral health. This result was unexpected, because some studies^16^^,^^17^^,^^19^^,^^23^^,^^27^^,^^30^^,^^31^^,^^32^^,^^34^ have shown that RCTs within oral health were of poor or inadequate quality.

Similarly, Peters (2015) assessed the quality of reports and abstracts of RCTs within the literature relating to otorhinolaryngology and found that the quality of reporting of RCTs was suboptimal. This author showed that these articles did not report the allocation process sufficiently.

The only study comparing medicine with dentistry was by Sjögren,^17^ yet the scale that this author used to evaluate RCTs was the Jadad quality assessment scale. Moreover, the sample was too small: only 200 in each field. The allocation implementation process, which included generation of a random allocation sequence and allocation concealment, was not simultaneous.

There is a dynamic movement towards enhancement of reports and specifically the quality of RCTs, within all fields.^16^^,^^17^^,^^18^^,^^19^^,^^20^^,^^21^^,^^22^^,^^23^^,^^24^^,^^25^^,^^26^^,^^27^^,^^28^^,^^29^^,^^30^^,^^31^^,^^32^^,^^33^^,^^34^^,^^35^^,^^36^^,^^37^^,^^38^ Some authors have shown that improvements in the methodology of published studies within the field of oral health have been achieved. Nonetheless, great concern remains regarding description and reporting of the methods used, in published RCTs.^27^^,^^32^

There was similarity between the fields regarding the methods used in the allocation process. Simple random allocation is the easiest and most basic approach towards providing unpredictability of treatment assignment. Good methods of generating random allocation sequences include using a random-numbers table or a computer software program to generate the random sequence. There are manual methods for achieving random allocation, such as tossing a coin, drawing lots or throwing dice. However, these manual methods in practice often become nonrandom, are difficult to implement and do not leave an audit trail.^31^

To achieve allocation concealment, opaque sealed envelopes were used in both groups. This method is usually considered acceptable, but it may be susceptible to manipulation. Central randomization is the preferred method.^18^

Randomization reduces bias in clinical trials and provides a basis for ensuring the validity of data analysis using statistical testing. It usually just requires a table of random numbers. Simple randomization is adequate for large trials, while block randomization is a method for balancing equal numbers of patients in each treatment group. Stratification allows balanced distribution of one or more confounding prognostic variables among treatment groups to ensure that the groups have similar prognoses. Block randomization and stratification improve the validity of trials with smaller numbers of patients. Computer software facilitates randomization.^29^ Shulz^13^^,^^15^ found that none of the restricted randomization approaches that were used for generating allocation sequences, regardless of their complexity and sophistication, were better than simple unrestricted allocation for prevention of bias.

In relation to sequence and allocation concealment, Shulz^13^^,^^15^ showed that deciphering does occur, most commonly because the method of allocation concealment was inadequate. Even though Altman^14^ showed that carrying out allocation concealment is a very simple procedure that can be incorporated into the design of any trial, the data of the present study showed that the dimension of allocation concealment is a limiting factor with regard to production of RCTs with low risk of bias. Thus, allocation concealment is the main barrier against production of RCTs with control over selection bias.

The search for the present analysis was made only in the PubMed database, and other medical databases were excluded. However, PubMed is one of the largest and most widely available databases: it is accessed through the National Institutes of Health (NIH).

## CONCLUSION

Efforts need to be made to focus on the allocation implementation process, using only a single step: generation of random allocation sequences and simultaneous implementation of the sequence in such a way that the allocation is concealed. The quality of reporting within the fields of oral health and ENT is suboptimal and needs to be improved so as to match the quality already attained in other fields of healthcare. It is of great importance to take these findings into account, in order to improve the level of evidence of future randomized controlled trials within all fields of healthcare.
